# Acute cellular rejection after heart transplantation and its remission visualized by cardiac magnetic resonance

**DOI:** 10.1093/ehjcr/ytab085

**Published:** 2021-02-28

**Authors:** Zhehao Dai, Masae Uehara, Eisuke Amiya, Masaru Hatano, Issei Komuro

**Affiliations:** Department of Cardiovascular Medicine, Graduate School of Medicine, The University of Tokyo, 7-3-1 Hongo, Bunkyo, Tokyo 113-8655, Japan

Recent advances of parametric mapping techniques have allowed cardiac magnetic resonance (CMR) to effectively detect acute cellular rejection (ACR) after heart transplantation. Herein, we share CMR findings in two cases of ACR. A 45-year-old man who underwent orthotopic heart transplantation for anthracycline-induced cardiomyopathy was scheduled for a surveillance biopsy at week 12 post-transplantation. Histopathologic findings of his right ventricular endomyocardial biopsy revealed multiple foci of lymphocyte infiltration with myocyte damage [International Society of Health and Lung Transplantation (ISHLT) Grade 2R, *Panel A*]. CMR showed diffusely prolonged native T1 (1345 ± 43 ms, *Panel B*) and T2 (53.7 ± 1.4 ms) values. We treated him with methylprednisolone 1000 mg daily for 3 days followed by a tapered regimen, namely prednisolone 25 mg daily for 2 days, 20 mg daily for 2 days, and 15 mg daily for maintenance at discharge. Eight days after the first biopsy, his follow-up CMR showed reduced native T1 (1298 ± 45 ms, *Panel C*) and T2 (50.8 ± 1.1 ms; T2-weighted images and native T2 mapping; see Supplementary material online, *Figure S1*) values, and biopsy revealed no acute rejection (ISHLT Grade 0, *Panel D*). Similarly, a 43-year-old man who underwent orthotopic heart transplantation for ischaemic cardiomyopathy presented with his 7th year’s surveillance biopsy demonstrating ACR of ISHLT Grade 2R, and his CMR showing diffusely prolonged native T1 (1494 ± 30 ms, *Panel E*) and T2 (68.2 ± 3.5 ms) values. Both CMR (native T1 value 1416 ± 59 ms, *Panel F*; native T2 value 57.7 ± 4.3 ms) and histopathological findings (ISHLT Grade 0) improved after intravenous corticosteroid treatment (Histology, T2-weighted images, and native T2 mapping; see Supplementary material online, *Figure S2*). CMR with parametric mappings might contribute to non-invasively diagnosing ACR in cardiac allograft recipients.

**Figure:**
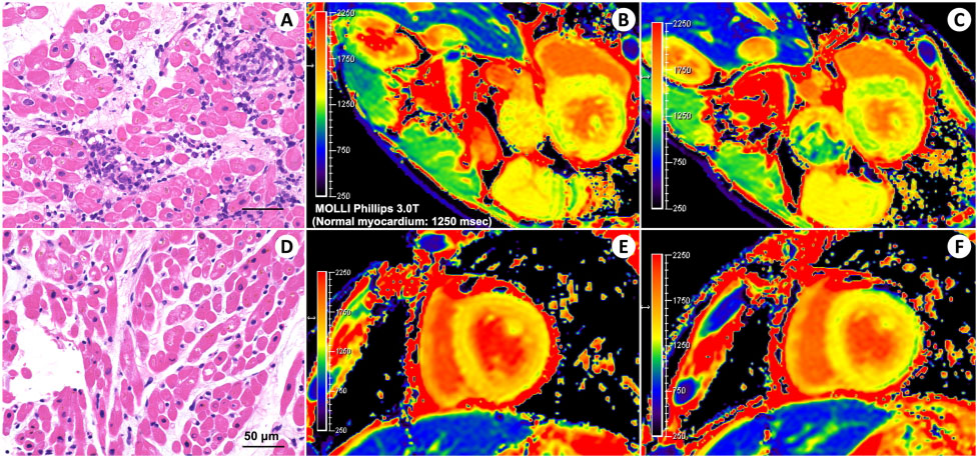


## Supplementary material

Supplementary material is available at *European Heart Journal - Case Reports* online.

**Consent:** The authors confirm that written consent for submission and publication of this case report including images and associated text has been obtained from the patient in line with COPE guidance.

**Conflict of interest:** None declared.

**Funding:** None declared.

## Supplementary Material

ytab085_Supplementary_Data

