# Supplementary figures and images for: Acute cellular rejection after heart transplantation and its remission visualized by cardiac magnetic resonance
Source: Eur Heart J Case Rep. 2021 Feb 28;5(3):ytab085. doi: 10.1093/ehjcr/ytab085 (PMC7936919; doi:10.1093/ehjcr/ytab085)

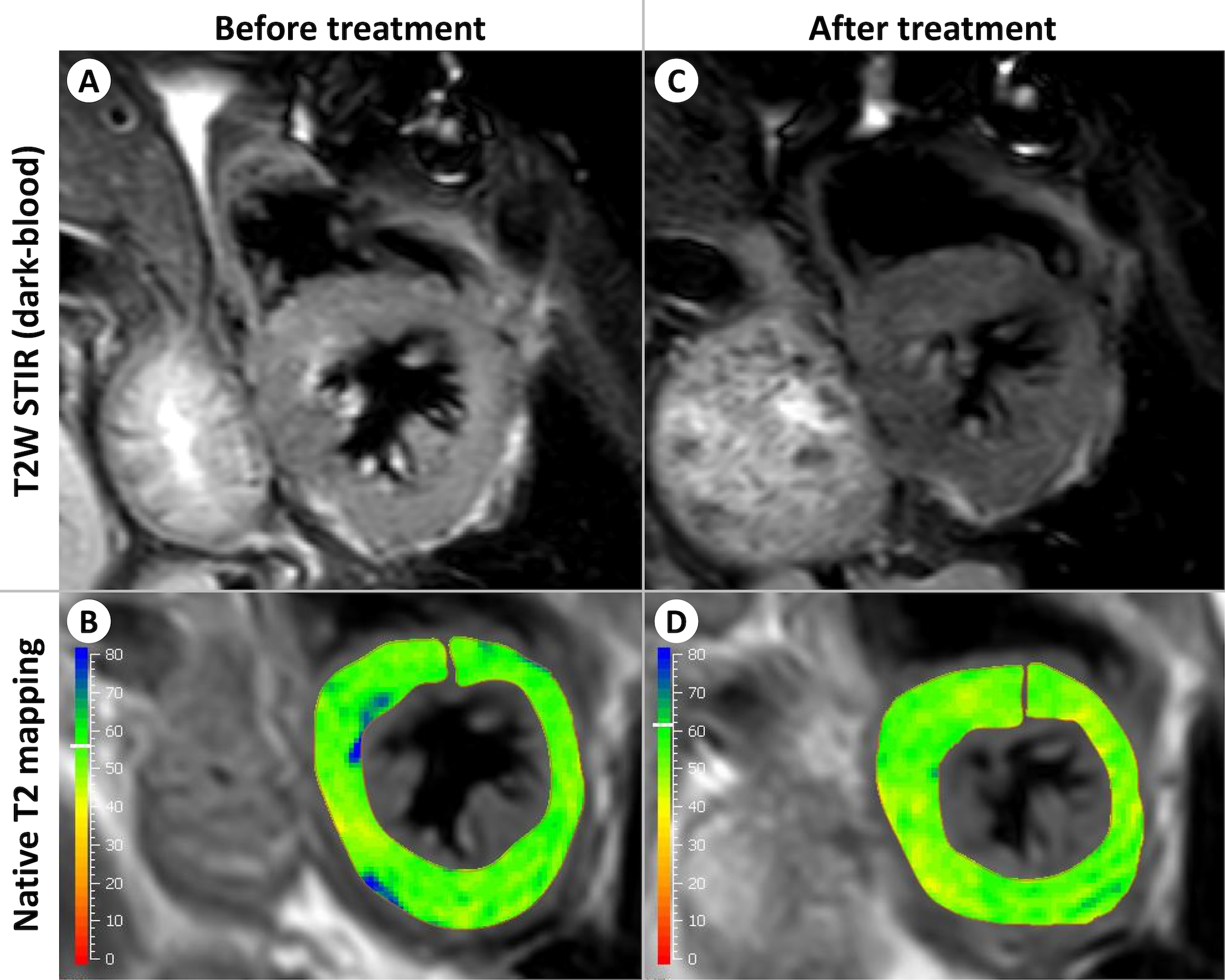

Supplement: ytab085_Supplementary_Data [file ytab085_supplementary_data.zip › ytab085_Supplementary_Data/Figure S1.tiff]

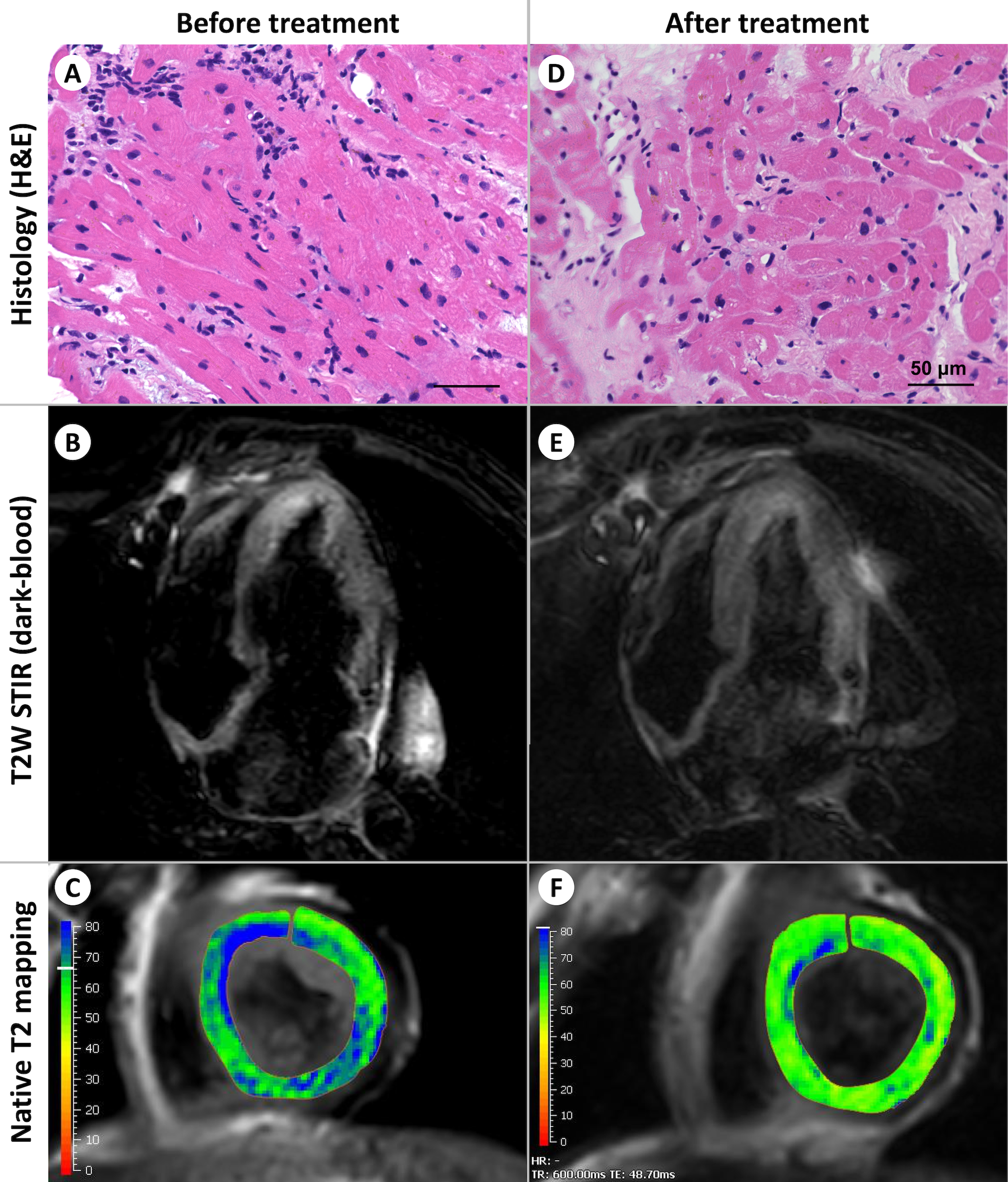

Supplement: ytab085_Supplementary_Data [file ytab085_supplementary_data.zip › ytab085_Supplementary_Data/Figure S2.tiff]
